# Surgical Intensity and Specialization Preferences in Healthcare: An Operation- and Process-Management Perspective Using Bibliometric Analysis, Cognitive Mapping and Analytic Network Process (ANP)

**DOI:** 10.3390/healthcare14111552

**Published:** 2026-06-02

**Authors:** Yasemin Kılıç, Irem Duzdar, Oumayma Hamlaoui, Hakan Tozan, Mohammed Ait El Fqih

**Affiliations:** 1 Department of Industrial Engineering, Düzce University, Düzce 81620, Türkiye; yasminnklcc@gmail.com (Y.K.); iremduzdar@duzce.edu.tr (I.D.); 2 College of Engineering and Technology, American University of the Middle East, Egaila 54200, Kuwait; hakan.tozan@aum.edu.kw; 3 Ingénierie des Systèmes Intelligents, Industrielle et Mécanique (LISIME), École Nationale Supérieure D’arts Et Métiers Casablanca ENSAM, Hassan II University of Casablanca, Casablanca 20360, Morocco; simohammed.aitelfqih@univh2c.ma

**Keywords:** bibliometric analysis, co-occurrence network, cognitive mapping, Analytic Network Process (ANP), surgical intensity, specialty choice, workforce planning

## Abstract

**Background:** Surgical operations are an integral part of healthcare delivery and impose a substantial clinical and operational burden. Understanding how the operation- and process-management literature in healthcare reflects the intensity of surgical services and how this may affect the specialization preferences of healthcare professionals is important for strategic workforce planning. **Methods:** A bibliometric analysis was conducted on 272 academic publications obtained from the Web of Science Core Collection with the keywords “lean philosophy”, “health” and “process” to capture the operational and process-improvement perspective of healthcare services. In this work, the “lean philosophy” keyword was taken to denote the operation- and process-management view of healthcare services, not to reflect the whole literature on surgical intensity. This selection was performed due to the multiple reasons, with an example being that lean-related studies often discuss complexities of workflow, efficiency, organizational responsiveness, and quality optimization, which are aspects also directly linked to surgical operational intensity. The data were analyzed using the bibliometrix R package, R-4.6.0 to construct the keyword co-occurrence network. Based on this network, a cognitive map was designed to visualize the conceptual relationships among the themes. Thematic clusters based on the co-occurrence network were then evaluated and prioritized by using the Analytic Network Process (ANP). Pairwise comparison data were derived from seven experts (surgeons and healthcare managers), and the model was implemented in Super Decisions with consistency ratios below 0.10. **Results:** The findings of the co-occurrence analysis are five main thematic clusters with surgical intensity themes including Healthcare Services, Quality, Care, Health and Outcomes. The cognitive map shows that Healthcare Services and Quality have the most central positions and structural hubs in the literature, whereas Outcomes is a dimension of great importance in terms of performance. The ANP results show that Quality (limiting weight ≈ 0.21), General Topics (≈0.14) and Management and Leadership (≈0.13) are the most influential sub-themes with regard to surgical operational intensity and, indirectly, to specialization preferences. **Conclusions:** The findings reveal that quality management, organizational leadership and larger health policy concerns are closely associated with the intensity of operations of surgical services as depicted in the operation- and process-management literature. Healthcare workers might be inclined to relocate to job positions related to quality improvement and leadership in lieu of places with a high direct clinical burden. Such insights can guide the policies of strategic human resource planning and specialization balancing in healthcare systems.

## 1. Introduction

The healthcare sector is a dynamic and complex system that needs to be constantly adapted to growing patient demands, changing technologies, and limited resources. Within this system, surgical operations are one of the most important parts, both in the clinical and operational aspects, with important implications for patient outcomes, costs, and staff well-being. Surgery accounts for 30% of global burden of disease, and every year, approximately 310 million people have surgery worldwide, with demand continuing to rise due to factors such as aging populations and increasing patient morbidity [1,2,3,4]. Surgical services are also economically important and account for up to two-thirds of hospital revenue, approximately 60% of hospital income, and 40% of what patients spend [4,5,6].

Planning, scheduling and managing surgical services have direct implications for patient outcomes, cost and staff well-being. Accurately analyzing operational intensity, i.e., how often, where, and under what conditions surgical procedures are carried out, plays a crucial role in improving the quality of services, ensuring efficient use of resources and managing human capital effectively. In the current paper, the concept of operational intensity is considered in the context of its representation and thematic organization in the academic literature, not in terms of empirical measures of surgical workload or the volume of procedures. Surgical operational intensity in this case is a general term that can be used to describe clinical, organizational, and workload-related demands of surgical services, as found in the literature. In the bibliometric analysis, this construct is thus operationalized indirectly as a topic proxy in the form of recurring themes about healthcare operations, process complexity, quality management, organizational burden, and outcomes in surgical service settings. Healthcare systems struggle with chronic problems of managing surgical wait lists, making processes more efficient, and reducing cancellations and delays. These issues are worsened by growing demand, scarce resources, and clinical complexity, especially in public hospitals serving vulnerable populations [1,2,4,7]. Prolonged surgical delays can increase clinical risk, adversely affect patient outcomes and increase institution costs [3,7] and operational inefficiencies, including poor management of operating rooms and supply chains, adding to financial losses and poor patient experience [1,2,8].

Delays and inefficiencies in surgical services have direct consequences on the health of patients and their recovery. Major complications, including infections or organ injuries, can triple in-hospital stays, raise readmission rates and increase the chance of patients returning home with long-term effects on functional status and quality of life [1,3]. Fragmented communications and inconsistent care pathways throughout the perioperative continuum can result in confusion, poor satisfaction and unnecessary cancellations or readmissions [1].

Limited infrastructure and workforce, and supply chain challenges are common challenges in delivering effective surgical care. These challenges are particularly acute in low- and middle-income countries, where access to surgical care is limited for up to two-thirds of the world population, resulting in life-threatening complications and chronic disabilities [8,9,10,11]. Workforce shortages, overwhelmed operative capacity and inadequate infrastructure are further obstacles to timely and safe surgical interventions [2,9,10].

Beyond aspects of operation, the intensity of surgery may also influence the career paths of healthcare professionals. High workload, time pressure and complexity can cause some areas of surgical practice to be less appealing over time, with other areas that offer more opportunities for leadership, quality improvement or more controllable workload coming to the fore. These demanding aspects have often been invoked as barriers, with many individuals preferring specialties with a more controllable workload or work–life balance [12,13,14,15,16]. For example, long hours and frequent call shifts during surgical training are linked with reduced enthusiasm for pursuing a surgical career, and work hour limitations have been linked with increased perceptions of work–life balance [12,13,14]. The demanding nature of surgery, with the prevalence of burnout and mental health disorders experienced by surgical residents, is a further factor in career reconsideration or attrition [12,14,15].

As a result of the intensity and demands of surgical practice, some healthcare professionals are attracted to areas where they can have more controllable workloads and opportunities for leadership or quality improvements. In this case, research should be read as a literature-based framing of potential tendencies that might be related to a specialization and not an actual movement of professionals. For example, an estimated 5–10% of core surgical trainees leave their training and work in different careers (e.g., anesthesia, radiology, obstetrics and gynecology, and general practice, which may have more favorable work–life balance or other opportunities that are not available in surgical training) [16]. Additionally, the skills and traits that are developed in surgery such as leadership and decision-making skills are also transferable to executive or leadership roles within healthcare organizations, giving surgeons the opportunity to make a difference in healthcare beyond individual patient care [17,18,19,20].

Understanding how the academic literature conceptualizes these issues can offer valuable information about the potential determinant of specialization choices.

Multiple studies that identify and categorize the main research themes in surgical operations and healthcare processes have been conducted from the bibliography and thematic analysis perspectives. For example, theme clusters in anesthesiology and pain journals are robot-assisted surgery, perioperative care, pain management, regional anesthesia, cardiothoracic anesthesia, and neuroanesthesia, which reflect the alignment of clinical publications with practical anesthetic challenges and perioperative optimization [21]. In the field of robotic surgery research in general, keyword analysis has identified clusters such as applications and techniques of robotic surgery, urological surgery and complications, gastrointestinal diseases and interventions, robotic thyroid surgery, gynecological procedures, Da Vinci robot training, and pulmonary surgeries [22]. Similarly, in terms of the robotic surgery literature, eight successful keyword clusters were identified, with “robot-assisted surgery” and “minimally invasive surgery” as primary terms [23]. Thematic mapping has also been used for articles on AI and robotics in surgery, creating such categories as surgical performance enhancement, healthcare system integration and economics, and technical and architectural innovations. This mapping offers a holistic interpretation of the roles that AI and robotics play in modern surgical practice, stressing clinical, technological and political implications [24].

Several analyses have been done to create co-occurrence networks involving keywords to visualize concept relationships and thematic evolution. For example, in the area of remote robotic surgery (RRS), keyword co-occurrence and the clustering of terms identified five thematic domains: robotic systems, integration of telemedicine, surgical innovations, artificial intelligence (AI)-assisted imaging and 5G-enabled connectivity. Thematic mapping, with the help of clustering algorithms to visualize the conceptual structures and developmental trajectories, span from mere simulation and telepresence to intelligent convergence with deep learning and ultra-low-latency communication [25]. Thematic mapping in other studies has shown much the same about the emergence of AI and deep learning as dominant themes in recent years [26].

While the Analytic Network Process (ANP) is mentioned in the query as a method for prioritizing thematic clusters based on expert judgment, the following paragraphs do not make explicit reference to the use of ANP. However, there is a number of studies that do report the use of expert-driven thematic analysis and mapping to set research priorities and visualize evidence gaps. For example, concept maps and thematic frameworks have been created, to visualize evidence and show gaps in surgical research with studies spanning multiple themes included in all relevant categories [27]. Thematic analysis has also been employed to aggregate questions and synthesize findings into clinically useful categories, to support patient safety and decision making [28,29,30,31].

Several studies highlight the importance of expert opinion and bibliometric indicators in determining the relevance and impact of thematic clusters. For example, citation performance, author productivity and institutional contributions are used to assess the prominence of research themes and make strategic decisions in the development of journals and focus on research [22,32]. The combination of expert judgment and bibliometric data provides the basis for the prioritization of research areas and the discovery of emerging trends, such as the growing focus on the integration of AI in robotic surgery [22,26].

Across the reviewed literature, there is agreed application of bibliometric methods, keyword co-occurrence analysis, and thematic mapping to determine, visualize, and prioritize research themes within surgical operations and healthcare processes. The studies are in agreement on the value of these methods for the identification of research hotspots, the tracking of thematic evolution and the support of strategic decision making in both clinical and technological areas [22,24,25,26,32,33]. There is also acknowledgment of a general sense of the increasing need for interdisciplinary integration, especially the intersection of surgery, engineering and computer science, as seen through the themes of AI and deep learning being dominant [22,25,26].

In our work, we examine the academic development of research on the intensity of surgical operations and its possible impact on the choice of specialization in healthcare. We take a holistic and methodological approach that involves a combination of bibliometric analysis, cognitive mapping and the Analytic Network Process (ANP). First, we analyze publications indexed on the Web of Science database to identify thematic structures related to surgical operations and healthcare processes. Second, we build a co-occurrence network of keywords and extract a cognitive map to visualize the conceptual relationships. Finally, we use the ANP to set the priority of thematic clusters on the basis of expert judgments and to quantify the relative importance of the thematic clusters in determining operational intensity and specialization preferences. Bibliometric analysis, therefore, detects the thematic organization of the literature, cognitive mapping enables us to determine the conceptual relationships among the themes, and the ANP ranks the relative importance of these themes based on expert judgment. The analysis is undertaken based on the literature concerning lean and process improvement in the field of healthcare, which is one of the operational lenses of surgical intensity and its organizational-wide consequences.

While past research has employed bibliometric methods to trace research trends in the field of healthcare and has employed the ANP in strategic decision making and quality evaluation, the combination of these methods to specifically explore surgery intensity and its implications for specialization preference is limited. To the best of our knowledge, this is among the first studies to combine bibliometric co-occurrence analysis, cognitive mapping, and the ANP for examining the conceptual and decision-oriented structure of this topic.

The study makes a three-fold contribution: methodologically, this study is a synthesis of bibliometric analysis, cognitive mapping, and the ANP to organize and rank the literature; theoretically, this study is an interpretive framework relating surgical operational intensity to workforce planning and specialization policy in healthcare systems; and practically, this study clarifies implications for workforce planning and specialization policy. On a bigger scale, the current research study will be a complement to workforce analytics and health human resources modeling because it provides a literature-organized and expert-focused opinion on the factors which may be of interest to specialization planning.

## 2. Materials and Methods

### 2.1. Data Source and Search Strategy

The bibliometric analysis was performed on the Web of Science (WoS) Core Collection database. The search was focused on the literature related to the intersection of lean approaches, healthcare services and related processes (with a particular interest in surgical operations and associated workflows). The following keywords were used: “lean philosophy”, “health” and “process”. The search was limited to the time period 2008–2024, and the search date was 25 May 2024. The year 2008 was used as a starting point to concentrate the analysis on the more current literature on healthcare process improvement and operation management, where the themes have more reliably been reflected.

The initial query involved research articles and review articles that were written in English. Publications that are clearly not related to healthcare services or operations (for example, dealing with non-health sectors) were excluded. The determination of relevance was made in regards to the degree to which the title, abstract and, where applicable, the entire record revealed a substantive focus on healthcare operations, service processes, organizational management or surgery-related workflow situations. After screening and eligibility checks, there were 272 academic publications that remained for analysis. Consequently, the current dataset may be interpreted as an operation-management subset of the overall literature regarding the subject of surgical workload and specialization preferences.

### 2.2. Study Selection and PRISMA Flow

The process of selecting the studies was based on an approach similar to PRISMA to guarantee transparency on the identification and filtering of documents. The steps were as follows:

1. Identification: Records were identified in the Web of Science database using the specified keywords and filters (document type, language and time span).
2. Screening: Titles and abstracts were screened in order to exclude records that were unrelated to healthcare or operational processes.
3. Eligibility: Full texts or detailed abstracts were reviewed, if needed, to ensure the relevancy to surgical operations, healthcare processes, and associated management aspects.
4. Inclusion: A final group of 272 publications were included in the bibliometric analysis.

**Figure 1:**
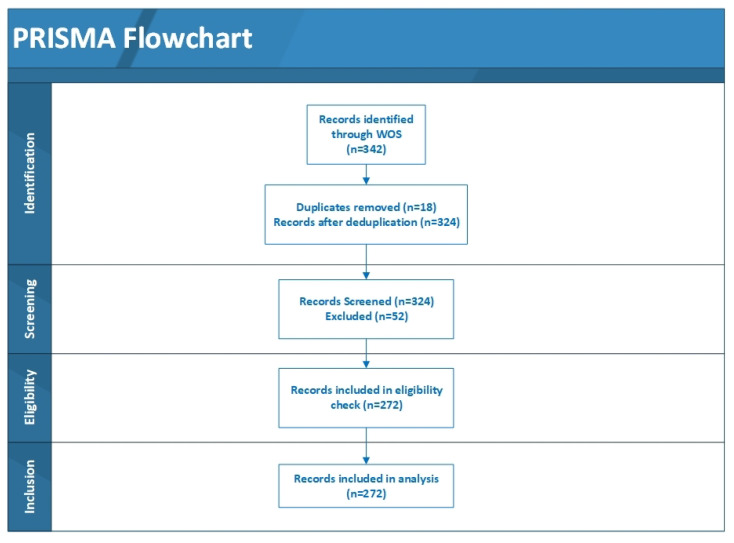
PRISMA flow diagram of the bibliometric data selection process.

The flow of records through these stages is summarized in Figure 1, which includes a PRISMA-style diagram that illustrates the number of records at each step, reasons for exclusion and the final dataset that was used for analysis. To be more precise, the screening procedure was performed in the following order: initially, records were determined based on the predefined Web of Science query and filters, followed by the screening of records at the title and abstract level based on relevance to healthcare operations and associated process themes; eventually, eligibility was evaluated regarding the scope of a study before the latter was included in the final bibliometric dataset. Therefore, the values in the flowchart indicate a progressive reduction of the dataset, not the counts of different stages.

### 2.3. Bibliometric Analysis and Co-Occurrence Network Construction

The bibliometric analysis was conducted with the use of the R-based Bibliometrix package. This tool is used to support descriptive bibliometrics and science mapping such as analysis of publication trends, co-authorship patterns and keyword co-occurrence networks.

In this study, the focus was on keyword co-occurrence, which makes it possible to identify thematic clusters and to identify the conceptual structure of a field. For that reason, the bibliometric stage was specifically focused on theme structuring through the co-occurrence of documents, while other higher-order-level indicators (such as the analysis of theme evolution, burst detection, bibliographic coupling and co-citation analysis) remained outside the scope of the present framework. Author keywords and, where available, Keywords Plus were extracted from the 272 publications. A co-occurrence matrix that measured the frequency at which pairs of keywords occurred in the documents was then created. Bibliometric histories and the keyword materials that were extracted to compute this matrix can be availed upon reasonable request to aid in the reproducibility of the mapping process.

**Figure 2:**
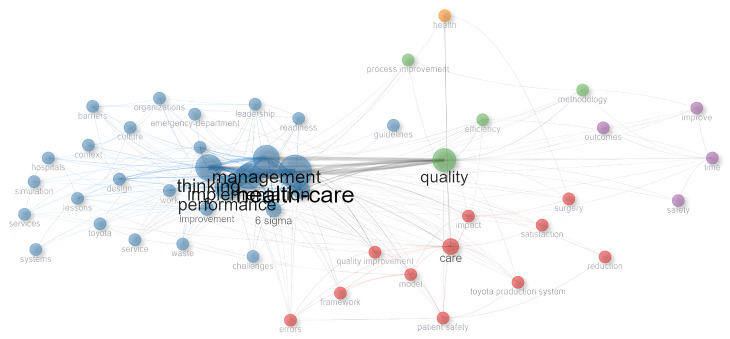
Co-occurrence network analysis of the healthcare-related surgical operations literature.

**Figure 3:**
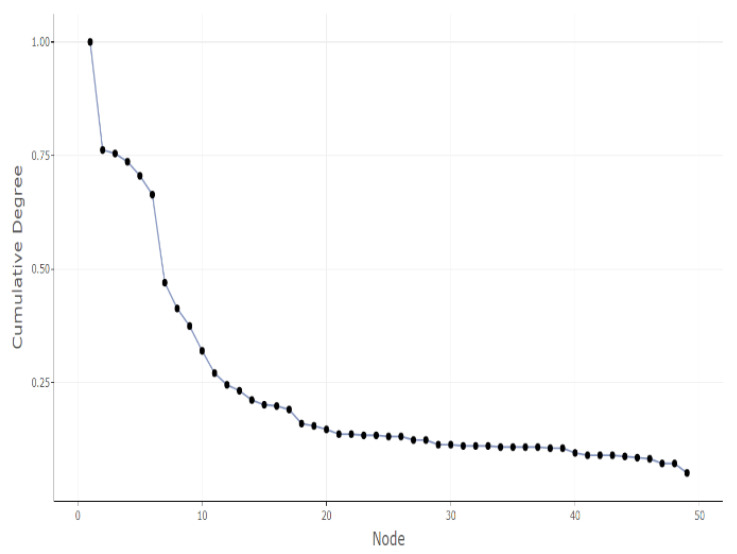
Cumulative degree distribution of keywords in the co-occurrence network.

The noise due to isolated terms was minimized by a minimum-frequency cutoff of two co-occurrences, and more structurally meaningful concepts were selected in the co-occurrence network. Although this criterion enhances the interpretability of the network, it can also decrease the appearance of emergent or niche themes that are less common but can become applied in the future to specialization patterns. The resulting network was analyzed in order to identify communities of closely related keywords, which were interpreted as thematic clusters. The clustering routine that is available in the Bibliometrix-based co-occurrence mapping workflow was used to perform community detection. The clusters were coded using different colors (e.g., blue, green, red, yellow, and purple) and labeled based on the dominant concepts that were found in a cluster (e.g., Healthcare Services, Quality, Care, Health, and Outcomes). The assigning of cluster labels was done in an interpretive manner depending on the recurring popular concepts in each community and must thus be perceived as analytic descriptions and not categories. The co-occurrence network was visualized in order to illustrate these clusters and their interconnections. The co-occurrence network was visualized to illustrate these clusters and their interconnections, as shown in Figure 2. The cumulative degree distribution, illustrating the proportion of highly connected keywords and highlighting central concepts shaping the literature, can also be visualized in Figure 3. Figure 3 is presented in such a way so as to be descriptively interpreted to understand the concentration of connectivity and is not meant to be a formal statistical test of scale-free behavior.

### 2.4. Cognitive Mapping

To gain a deeper insight into conceptual relationships among the identified themes, a cognitive map was created using CmapTools 6.04 software. Cognitive maps are visual representations that present concepts in the form of nodes and relationships in the form of directed links, thus affording them to depict semantic coherence and literature-based influence pathways among themes.

In this study, the major clusters that were derived from the co-occurrence network, with their representative sub-themes (e.g., Hospitals, Services, Working Conditions and Personnel, Management and Leadership, Process Improvement, Efficiency, Methodology, Quality Improvement, Outcomes Improvement, and General Topics), are represented as nodes in the cognitive map. The relationships between nodes are representative of the co-occurrence relationships as well as the interpretive synthesis of the authors on how these concepts are related in the literature. These directed links are not to be taken as formal causation but as conceptual affect and thematic direction, such as the association of Healthcare Services with Quality or the association of Quality with Outcomes. Practically, edges in the cognitive map were developed where recurrent patterns of co-occurrence and thematic similarity implied that there was some conceptual connection between clusters or sub-themes. Once the first map had been built, the general structure was conceptually checked internally against conceptual consistency and alignment with the underlying bibliometric patterns, in order to minimize arbitrary linkage. Though no formal inter-rater agreement procedure was implemented, this was done to enhance transparency and stability of the interpretive mapping procedure.

**Figure 4:**
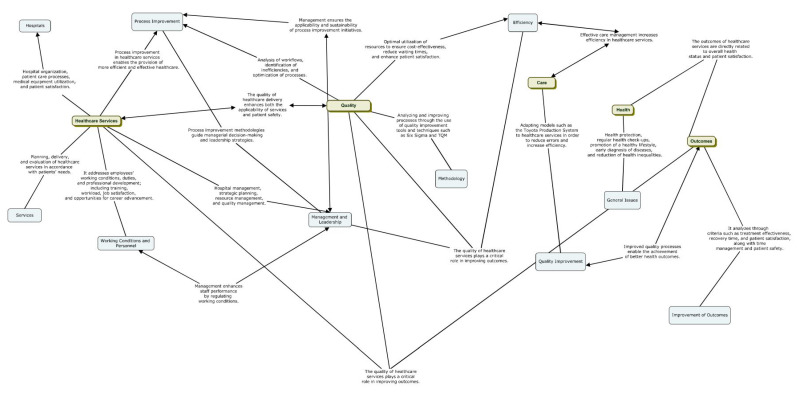
Cognitive map of thematic clusters and sub-themes related to surgical operations and healthcare services.

The resulting cognitive map in Figure 4 gives a holistic view of the connections among organizational structures, quality initiatives, care practices and public health issues, and outcome measures in the context of surgical operations and healthcare services.

Based on this, the cognitive map is to be understood as a conceptual and organizing device that is based on bibliometric tendencies, not as a validated causal model. Any apparent feedback relationships in the cognitive map should therefore be interpreted as conceptually inferred thematic connections rather than empirically validated feedback mechanisms.

### 2.5. ANP Model Structure and Expert Elicitation

The Analytic Network Process (ANP) was employed to prioritize themes and sub-themes of the cognitive map and to assess their relative influence over the intensity of operation and specialization preferences for surgery. The ANP was chosen due to the study design that presupposed interdependence between the themes and sub-themes, which necessitated a network-based prioritization framework compared with a strictly hierarchical one.

An ANP model in which higher-level decision components were clusters, such as Healthcare Services, Quality, Care, Health and Outcomes, while the sub-themes of these clusters (e.g., Hospitals, Services, Working Conditions and Staff, Management and Leadership, Process Improvement, Efficiency, Methodology, Quality Improvement, Outcomes Improvement, General Topics) were represented as nodes in the clusters was created. The overall objective of the model was to determine what factors have the greatest influence on the intensity of surgical operations and, by extension, on the choice of specialization of healthcare professionals. The dependency structure that is included within the ANP model, therefore, can be interpreted as conceptually based upon the cognitive map and expert-based judgment and not as a validated system of relationships.

Pairwise comparison data were obtained from seven experts having significant experience in surgery and/or healthcare management. These experts were chosen according to their professional experience and knowledge of the process of operational decision making in healthcare institutions. The sampling was to encompass respondents who were directly acquainted with surgical services, healthcare operations or managerial decision making, in order to have ANP judgments that would represent both practical and organizational aspects. The panel was strategically constituted to contain both clinical and managerial opinions, so that the pairwise comparisons made would be representative of a wider picture of the intensity of surgery operations, and to minimize the risk of bias, which this study would have been exposed to in case it had one professional background only. Each expert completed pairwise comparisons using Saaty’s 1–9 scale to judge the relative importance of the nodes and clusters with respect to the goal of the model and interdependencies.

**Figure 5:**
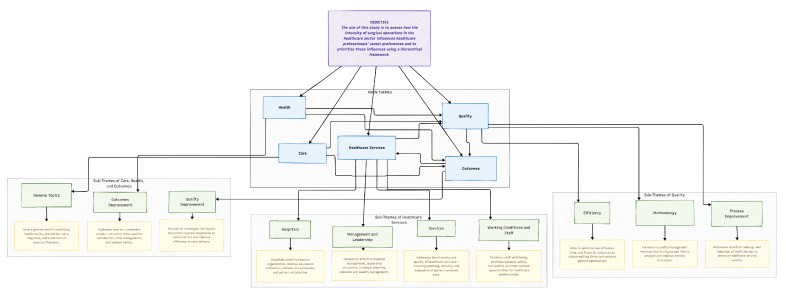
ANP structure for prioritizing themes related to surgical intensity and specialization preferences.

Individual judgments were combined with the use of the geometric mean. Despite the fact that the aggregation of judgments was done with the help of the geometric mean, the differences between the experts were not considered separately and could be addressed in further studies. The ANP model was in Super Decisions software V3.2. For each of the comparison matrices, the consistency ratio (CR) was calculated, and a threshold of CR < 0.10 was used to ensure acceptable consistency in judgments. The condition was met by all matrices. To conserve space, individual ratios of matrix consistency are not tabulated here, but all the pairwise comparison matrices met the agreed CR < 0.10 ratio. This was done to justify the inner consistency and reasonable reliability of the combined expert opinions in the ANP model. The resulting ANP model structure is illustrated in Figure 5, and the analysis of the consistency improvement and final priority weights are shown in the two tables in Section 3.3.

## 3. Results

### 3.1. Co-Occurrence Network Analysis

The co-occurrence network analysis grouped high-frequency co-occurring keywords to form different thematic clusters. Five prominent clusters were discovered in the healthcare services literature that are relevant to surgical operations:

- Healthcare Services (blue cluster);
- Quality (green cluster);
- Care (red cluster);
- Health (yellow cluster);
- Outcomes (purple cluster).

Within each cluster, sub-themes which were based on shared conceptual content and functional roles were defined. For example, under Healthcare Services, sub-themes were Hospitals, Services, Working Conditions and Personnel, and Management and Leadership. The Quality cluster included Process Improvement, Efficiency and Methodology, and the Care cluster included concepts related to Quality Improvement, patient safety, and models such as the Toyota Production System. The Health cluster was focused on general public health and preventative services, while the Outcome cluster was performance-oriented, with sub-themes such as Time and Safety.

These relationships are summarized in Table 1, which includes themes, sub-themes, descriptions and representative keywords. The clusters and the connections between them are graphically shown in Figure 2, with the colors representing the conceptual domains and the connections representing the co-occurrence relations.

The cumulative degree distribution in Figure 3 emphasizes the fact that a few nodes, such as “healthcare”, “management”, and “quality”, have high connectivity, which reflects their central role in structuring the field. Concepts with lower degree values have more peripheral positions and are indicators of specialized or niche topics. Overall, the network validates the importance of organizational and quality-related notions in the study of surgical operations and healthcare services. Centrality in the current research is understood mainly based on the connectivity structure that is observed in the network, whereas more detailed quantitative measures of centrality can be applied in later works.

### 3.2. Cognitive Map

The cognitive map was developed as a way of having a more nuanced understanding of the conceptual interactions among these thematic clusters. Each theme is a color-coded node, and sub-themes and explanatory components are connected by directed arrows.

On the left side of the map, the Healthcare Services theme (blue cluster) includes sub-themes of Hospitals, Services, Working Conditions and Personnel, and Management and Leadership. These sub-themes characterize organizational structures, mechanisms to deliver services, staff conditions and leadership configurations that determine the operational context of surgical services. The effectiveness of healthcare services is related to the theme of Quality, which shows how organizational structures and management practices affect process improvement, efficiency and methodology.

The Quality theme (green cluster), in the center of the map, appears to be a strategic connector, with influence on both Care and Outcomes. The sub-themes Process Improvement, Efficiency, and Methodology describe the initiatives to optimize the use of resources, minimize the number of mistakes, and enhance the performance of services. The combination of structured quality improvement methodologies, such as Six Sigma and Total Quality Management (TQM), is especially highlighted in this cluster.

On the mid-right-hand side of the map, the theme of Care (red cluster) is associated with quality-oriented strategies, such as quality improvement frameworks, and the application of the Toyota Production System to healthcare, which are aimed at reducing errors, improving patient safety, and improving efficiency. These ideas emphasize the human and service-focused aspects of healthcare delivery and are of particular interest is the surgical context.

At the top right, the Health theme (yellow cluster) covers the areas of public health and preventive services such as health promotion, regular screenings, lifestyle interventions and actions to reduce health disparities. The connection between this theme and General Topics highlights the wider policy and societal context in which a surgical services and specialization decisions are seen.

Finally, in the lower right-hand corner, the Outcomes theme (purple cluster) represents the performance dimension of healthcare services. It is focused on outcome improvement, treatment effectiveness, recovery times, time management and safety. This theme can be considered to be the aggregate reflection of the effects generated by Healthcare Services, Quality, Care, and Health.

Centrality analysis of the cognitive map shows that Quality and Healthcare Services are solid clusters that have many connections between them and act as a hub that influences other themes. Outcomes is also a strong theme based on key performance indicators. Care and Health has medium influence but supports functions in the system. General Topics and some of the peripheral sub-themes are illustrated as relatively weaker nodes with fewer connections, which have more complementary roles in the overall knowledge structure.

### 3.3. ANP Results

The themes and sub-themes from the cognitive map were organized into an ANP model to explore the relative importance of the themes. The structure of the ANP network is presented in Figure 5. Clusters are main themes, while nodes are sub-themes and related decision elements.

Pairwise comparisons from experts were combined and analyzed by SuperDecisions. Consistency indices for all matrices were less than 0.10, which suggests that the expert judgments were of acceptable coherence. The improvement in consistency analysis is summarized in Table 2, which contains, for each pair of criteria, the original and optimized values and the percentage improvement in inconsistency.

The “%Improvement” column indicates how changes toward best values reduced inconsistency. Notable improvements were found for the pairs Quality–Health (35.15%) and Healthcare Services–Outcomes (35.13%), confirming substantial interdependencies between quality-related processes, health outcomes, and service configurations. Moderate improvements were observed for pairs such as Quality–Healthcare Services and Health–Outcomes, highlighting the structural coherence of the ANP model.

The final priority weights are given in Table 3. According to the limiting values, the most important sub-themes are Quality (limiting weight ≈ 0.2105), General Topics (≈0.1401), and Management and Leadership (≈0.1269). Meanwhile, this prominence, in part, might be characteristic of the organization of the retrieved corpus and the operational-process approach to the study and should be construed in that analytical domain. Other important sub-themes include Healthcare Services (≈0.1933), Outcomes (≈0.0439), Efficiency, Methodology, Process Improvement, Hospitals, Services, and Working Conditions and Staff. The normalized-by-cluster value of General Topics is large, indicating its relative domination in its own cluster, and the limiting weights indicate that the entire network is determined by both systemic and operationally oriented themes like Quality and Healthcare Services.

These results suggest that these factors are of central importance in the development of operational intensity of surgery and thus the health policy, contextual factors and organizational leadership in determining specialization preferences. A more refined quantitative analysis of pairwise sub-theme interactions outside the existing priority interpretation would be a valuable addition to the existing ANP analysis.

## 4. Discussion

This study offers a comprehensive overview of the organization of research studies dealing with surgical operations, healthcare processes, and other management issues in the academic literature and how they may be related to specialization preferences. With this, the bibliometric methodology used in this case is no longer a measure of operational intensity as a clinical reality, but a question of the representation of intensity-related themes, their interaction, and their priority levels in the literature. In theory, this study proposes that the concept of surgical operational intensity can be viewed not only as an issue based on the workload but also as a literature-organizational, quality, and policy-structured interaction.

Research studies in the academic literature focusing on surgical operations, healthcare processes and issues in management are organized by some of the major themes and methodologies. These research studies tend to have a focus on the operational and organizational variables, the mechanisms of operational causal pathways, and economic and managerial implications for delivering healthcare. The literature is usually organized by themes such as the role of operational variables, volume, patient routing, error management, process improvement, etc. There is also an increasing focus on future directions of research, such as personalized medicine, value-based healthcare and connected health, with calls for greater interaction between operations management and the medical community to help improve healthcare delivery [34,35,36,37]. A great range of operations research (OR) and operations management (OM) techniques are applied in healthcare management sciences, which might reflect some orientation for some specialization preferences. Taxonomies have been created to offer common terminology and classification mechanisms for these methodologies with articles classified in order to exhibit the descriptive power of these methodologies. Quantitative and qualitative approaches are applied to find out the investigation themes, methodological trends, and future research paths. Simulation and modeling, including discrete-event simulation, are often used to optimize resources, improve patient flow and reduce costs in healthcare settings, which has created accessible resources for healthcare improvement, directing preference towards some specific specializations [36,37,38,39].

The co-occurrence network shows that concepts related to Healthcare Services and Quality are very central, hinting at the prevalence of organizational structures and quality-related initiatives through which surgical operations are studied. Similar results indicate that safety and quality are identified as key issues in healthcare delivery, particularly in the context of surgery, which is now being acknowledged as having a global impact on health outcomes [40,41,42]. Surgical complications are a major cause of morbidity and mortality, with at least 50% of these complications being preventable, which makes it important to highlight the need to invest in strong safety culture and patient safety initiatives aligned with healthcare services and quality management necessity [41,42,43]. Results from the literature have also shown that the provision of quality surgical care is central to the surgeon–patient relationship and that quality monitoring systems are important for evaluating healthcare processes and outcomes [44]. The complexity of operating room scheduling, with multiple entities involved (surgeons, nurses, ICU beds, etc.) has led to an increase in interest in optimizing these organizational processes [45]. Quality improvement programs such as the National Surgical Quality Improvement Program (NSQIP) have been implemented to facilitate quality data reviews and have resulted in a documented decrease in morbidity and cost saving [46,47]. The American College of Surgeons (ACS) and other organizations have developed quality efforts that focus on quality in surgery, which leads to better operative results and patient outcomes [47,48,49,50]. All the previous results reflect a specific trend for some preferred specializations, such us healthcare service, quality improvement and patient safety.

The cognitive map further illustrates that there are mediating factors between organizational arrangements (e.g., hospitals, management, and staffing) and outcomes (e.g., quality considerations), as well as care practices and public health issues (contextual layers).

The results found by the ANP further reinforce these observations by demonstrating that three factors are most influential in the network: Quality, General Topics, and Management and Leadership. Quality’s high priority relates to the increasing importance given to process optimization, safety and performance measurement in surgical services. The prominence of Management and Leadership indicates the importance of the structure of governance, strategic planning and resource management in managing surgical workload. General Topics, which encompasses more general health policy and public health aspects, emphasizes the role of systemic and contextual factors. These high interdependencies in the real world of surgery imply that surgeons feel the intensity of workload imposed not only by direct clinical demands but also by the quality structures and service organization that surrounds them, which in combination determine patient outcomes and sustainability of day-to-day practice. Surgical specialty preferences are determined by a set of interconnected drivers of quality and system/operational factors and management/leadership, where quality factors (process optimization, safety, and performance measurement) align with preferences for highly trained staff, lower complication risk, access to multidisciplinary teams with specialized expertise; governance structure and effective management influence the way in which services are organized and perceived, and practical issues, such as waiting time and access to specialized staff, have a significant impact on choices [51]. Besides these network-level aspects, there are also preferences based on the surgeon/institution reputation and technical expertise/certification [52,53,54,55], as well as mentorship and role models that bring exposure to surgery and motivate professionals [56,57,58,59]. Decisions are further influenced by perceived prestige and career opportunities [60,61,62]; lifestyle considerations (work hours, training lifestyle, and work–life balance), which are a source of discouragement or become less important depending on context [56,60,62]; and gender/social factors, where evidence shows differences in how strongly family compatibility and balance influence specialty choice [61,62,63,64,65,66].

From the point of view of specialization preference, these results indicate that healthcare professionals may be affected not only by clinical factors but also by expectations of quality, complexity of management, and organizational burden of the surgical fields. Nonetheless, the current bibliometric data lack the direct demonstration of actual professional migration; this implies an interpretive bias based on the thematic saliency of quality, leadership, and organizational variables in the literature. Specialties with high complication rates, demanding direct care and on-call schedules, may become less appealing in the long run, especially if they do not have good support from quality and leadership systems. On the other hand, roles that provide opportunities in quality improvement, strategic leadership and system-level planning may appeal to professionals who are looking to influence healthcare delivery beyond patient care.

Our results are consistent with broader discussions in the literature on surgical workforce sustainability, burnout and changing career preferences, in which workload intensity and organizational conditions are repeatedly mentioned as important determinants. Such results are also consistent with larger burnout and career-choice lenses, where the workload burden, work–life balance, perceived support, and opportunities of professional development influence specialty attractiveness. The integrated methodological approach adopted here (a combination of bibliometrics, cognitive mapping and the ANP) adds value by quantitatively relating conceptual structures in the literature and a decision-oriented prioritization framework [67].

### 4.1. Practical Implications

For policymakers and hospital administrators, the findings emphasize the importance of taking into account quality management and leadership capacity when addressing surgical workload and imbalances in specialization. These connotations are to be interpreted as literature-based and professional-advised decision-making recommendations as opposed to explicit predictive indications of workforce behavior. Planning initiatives include: enhancing leadership and management training for surgeons and healthcare professionals, introducing quality improvement roles into clinical teams, re-engineering processes to improve outcomes, minimizing avoidable workload, and using bibliometric and decision-analytic tools to monitor the co-evolution of research and practice. For example, with a shortage of recruitment in surgical specialties, hospital administrators might be able to infer that the high limiting weight of Quality suggests that a better-quality environment based on better-laid safety protocols, structured improvement programs, mentoring, and leadership opportunities might make these specialties more appealing and palatable. An ethical approach to any policy application of such findings should also be considered carefully, so that specialization planning can help in the support of professional autonomy and sustainability of the system, instead of becoming a coercive steering mechanism.

Such strategies may be useful in creating a more sustainable and attractive environment for surgical specialties. Future studies can further develop the current framework with scenario-based or policy-simulation applications that can translate ANP priorities into other workforce planning approaches.

### 4.2. Limitations and Future Research

There are several limitations of this study. First, it is only based on the Web of Science Core Collection, and it uses a particular set of keywords (i.e., “lean philosophy”, “health”, and “process”), which may not capture all relevant studies, which may be indexed in different databases or with different terminology. Moreover, coverage of the indexing of various databases including Scopus and PubMed can affect the exposure of relevant studies and thus must be taken into account when interpreting the current corpus. Since the literature reviewed is based on various countries and health-system contexts, the prevalence of themes may not be supposed to be universal across regional or institutional environments. To be more precise, the chosen search keywords were supposed to highlight the lean and process-improvement outlook behind the healthcare services, thus placing surgical intensity into the context of operations and management. Based on this, the result ought to be analyzed within this scope of analysis. Second, the cognitive map and ANP structure, while based on bibliometric data and expert judgment, are still dependent upon modeling decisions and the subjective inputs. Due to the same reason, the precise copying of the cognitive map and ANP structure can be inconsistent even among corpora, panel constructions, and analytical options, despite the fact that the overall analytical structure can be maintained. The current study did not perform a separate external expert validation round on the cognitive map, and this might enhance future replications of the framework. Also, as the stability check was not carried out by an independent coder, the exact configuration of some links in the cognitive map may be open to interpretive judgment and should be taken into account when assessing the strict reproducibility of the mapping results. Besides that, there is a preference to using a minimum co-occurrence threshold to focus on structurally determined themes, and recently developed or highly specialized topics might receive lower prominence. Third, the expert panel was made up of seven participants, which is acceptable for the ANP; nevertheless, it is a relatively small sample. Future studies can enhance further transparency by reporting other panel descriptors, like years of experience and distribution of specialties. Moreover, even though the consistency ratio threshold was used to determine the consistency of the judgment, a formal sensitivity analysis was not performed, which may be used in future research to further investigate the strength of the ANP prioritization outcomes. Therefore, the prioritization weights reported should be viewed as relatively stable within the current expert judgment framework, but they were not fully stability-tested using alternative sets of input weights, and small changes in the pairwise comparisons could alter the ranking of some sub-themes. Future research can expand the search by adding to the perioperative care query with surgery-specific terms, like operating room management, perioperative care, surgical workload, burnout, specialty choice, and career preference. More robustness tests, like bootstrapping or repeated-panel stability tests, can also be considered in further research to supplement the current consistency test. A comparison with other prioritization methods, like AHP or DEMATEL, can be used to evaluate methodological sensitivity in further extensions of the framework. In addition, other structuring and decision-support methods, such as ISM, can also be used for comparative benchmarking, which can offer another view of the robustness of the current interpretation based on the ANP.

Future studies could build on this work by employing additional bibliographic sources, optimizing the search strategy, and combining this work with empirical data on actual choices of specializations, measurement of workloads, and indicators of burnout. Mixed-methods studies combining bibliometric mapping, survey data from healthcare professionals, and sophisticated decision-analytic modeling could be used to create a more comprehensive understanding of the relationship between the intensity of surgery, career structures and workforce dynamics. Further bibliometric studies might also include quantitative measures of centrality, thematic evolution, burst analysis and coupling/co-citation analysis to enhance the structural interpretation of the literature.

## 5. Conclusions

This study examined the reflection of the intensity of surgical operations in the academic literature and their possible effects on specialization preferences of healthcare professionals using an integrated methodology framework. The results must be understood in the frames of the literature of the operation and process management embraced by the chosen search strategy. Using bibliometric analysis, a co-occurrence network, cognitive mapping and the ANP, the key thematic clusters related to Healthcare Services, Quality, Care, Health and Outcomes and the relative importance of these clusters were identified. The results show that operational intensity is not a simple concept or a numerical measure of workload but a multidimensional phenomenon that depends on quality management, organizational leadership, and greater health policy structures. The ANP findings suggest that Quality, General Topics, and Management and Leadership are the most influential sub-themes, pointing at the importance of dealing with both organizational and systematic aspects when planning surgical services and specialization policies. From a workforce perspective, the analysis suggests that intensive and outcome-sensitive surgical fields might be less attractive if not supported by strong quality and leadership frameworks, while domains that provide opportunities for quality-oriented and leadership roles might be preferred more and more. The results imply a potential preference of healthcare professionals for those professions related to quality improvement and leadership in case direct clinical burden is perceived to be high; this, however, must be assumed to be a literature-based implication but not a direct indication of professional migration. To be more precise, the current results are to be viewed as the reflection of the literature highlighting and relating these themes, not as the direct observation of workforce behavior. Ensuring sustainable and balanced healthcare delivery thus involves combining strategies for specialization planning with quality initiatives, leadership development, and workload management. The integrated approach proposed here can be used as a model for researchers and decision makers in their interest to integrate bibliometric findings with decision-analytic tools to inform strategic planning in healthcare systems.

## Figures and Tables

**Table 1:** Results of co-occurrence network analysis.

Theme	Sub-Theme	Description	Keywords
Health Services (blue cluster)	Hospitals	It examines hospitals as the core of health services, covering hospital organization, patient care processes, medical equipment usage, and patient satisfaction.	Hospitals, Services, Work, Management
	Services	It addresses the diversity and quality of health services, including the planning, delivery, and evaluation processes of healthcare services tailored to patients’ needs.	—
	Working Conditions and Personnel	It focuses on the working conditions, duties and professional development of employees in the healthcare sector; specifically, the emphasis is put on education, workload, job satisfaction and opportunities for career advancement.	—
	Management and Leadership	It examines the effective management and leadership structures of healthcare organizations, covering topics such as hospital administration, strategic planning, resource management, and quality management.	—
Quality (green cluster)	Process Improvement	It addresses process improvement to enhance quality in healthcare services, including workflow analysis, identification of inefficiencies, and efforts to optimize processes.	Process Improvement, Efficiency, Methodology
	Efficiency	It focuses on increasing efficiency in provision of healthcare services, involving optimal utilization of resources (e.g., human power, time, and cost), ensuring cost-effectiveness, lessening waiting times and increasing patient satisfaction.	—
	Methodology	It deals with the methods and techniques to enhance quality and efficiency in provision of healthcare services, including the analysis and improvement of processes by using quality improvement tools and techniques such as Six Sigma and Total Quality Management (TQM).	—
Care (red cluster)	Quality Improvement	It involves the use of strategies and frameworks to enhance the quality of care and to strive for adaptation of models such as the Toyota Production System to healthcare services, in an attempt to reduce errors and enhance efficiency.	Quality Improvement, Framework, Errors, Model, Toyota Production System, Impact, Surgery, Patient Safety, Reduction
Health (yellow cluster)	General Topics	It encompasses the general health status, quality of healthcare services, and public health issues of individuals, such as health protection, regular health screening, promotion of healthy lifestyles, early detection of diseases, and alleviation of health disparities.	Health
Outcomes (purple cluster)	Outcome Enhancement	It focuses on the evaluation of impact of treatment and care process in the patients; criteria like effectiveness of treatment, recovery time, patient satisfaction, time management, and patient safety are analyzed.	Outcomes, Improvement, Time, Safety

**Table 2:** ANP consistency analysis and pairwise comparison results.

Rank	Criterion A	Criterion B	Current Value	Best Value	Old Inconsistence	New Inconsistence	% Improvement
1	Quality	Health	2,000,000	1,128,685	0.053207	0.034514	35.15%
2	Healthcare Services	Outcomes	3,000,000	1,298,773	0.053207	0.034514	35.13%
3	Health	Healthcare Services	2,000,000	1,000,000	0.053207	0.040058	24.71%
4	Quality	Outcomes	2,000,000	3,747,847	0.053207	0.042085	20.90%
5	Care	Quality	2,000,000	1,096,364	0.053207	0.044980	15.46%
6	Care	Outcomes	3,000,000	4,972,264	0.053207	0.044980	15.46%
7	Quality	Healthcare Services	2,000,000	1,425,639	0.053207	0.049760	6.48%
8	Health	Outcomes	3,000,000	2,095,176	0.053207	0.049760	6.48%
9	Care	Healthcare Services	2,000,000	2,405,850	0.053207	0.051914	2.43%
10	Care	Health	2,000,000	1,550,036	0.053207	0.051914	2.43%

**Table 3:** ANP priority results.

Category	Normalized by Cluster	Limiting Weight
General Topics	0.84662	0.140078
Outcomes Improvement	0.02519	0.004168
Quality Improvement	0.12819	0.021209
Hospitals	0.11492	0.025690
Management and Leadership	0.56788	0.126950
Services	0.09714	0.021716
Working Conditions and Staff	0.22007	0.049196
Efficiency	0.40636	0.044854
Methodology	0.28307	0.031246
Process Improvement	0.31057	0.034281
Care	0.06164	0.030858
Health	0.04389	0.021970
Healthcare Services	0.38614	0.193308
Outcomes	0.08777	0.043940
Quality	0.42056	0.210538

## Data Availability

The dataset used for the bibliometric analysis are available from the corresponding author upon reasonable request.
